# Peptide Receptor Radionuclide Therapy with Lu-177-DOTATATE and Monitoring with Somatostatin Receptor PET/CT in Patients with Advanced Differentiated Thyroid Carcinoma

**DOI:** 10.1007/s11307-025-02053-w

**Published:** 2025-09-29

**Authors:** Sophie Carina Kunte, Vera U. Wenter, Adrien Holzgreve, Gabriel T. Sheikh, Liam Widjaja, Franz Josef Gildehaus, Simon Lindner, Ralf Schirrmacher, Christine Spitzweg, Christoph J. Auernhammer, Rudolf A. Werner, Mathias J. Zacherl

**Affiliations:** 1 Department of Nuclear Medicine, LMU University Hospital, LMU Munich Marchioninistr. 15, 81377 Munich, Germany; 2 Bayerisches Zentrum Für Krebsforschung (BZKF), Partner Site Munich, Munich, Germany; 3 Ahmanson Translational Theranostics Division, David Geffen School of Medicine at UCLA, Los Angeles, CA USA; 4 Department of Oncology, Division of Oncological Imaging, University of Alberta, Edmonton, AB Canada; 5 Department of Internal Medicine IV, LMU University Hospital, LMU Munich, Munich, Germany; 6 Division of Endocrinology, Diabetes, Metabolism and Nutrition, Adjunct Academic Appointment, Mayo Clinic Rochester, MN USA Rochester,; 7 German Cancer Consortium (DKTK), partner site Munich, a partnership between DKFZ and LMU and TUM Klinikum, Germany Munich,; 8 Interdisciplinary Center for Thyroid Carcinoma (ISKUM), LMU University Hospital, LMU Munich, Munich, Germany; 9 Department of Radiology and Radiological Sciences, Division of Nuclear Medicine, The Russell H Morgan, Johns Hopkins School of Medicine, Baltimore, U.S.A.

**Keywords:** Differentiated thyroid carcinoma (DTC), Somatostatin receptor (SSTR), PET, PRRT, Response, Lu-177-DOTATATE

## Abstract

**Purpose:**

Peptide receptor radionuclide therapy (PRRT) with Lu-177-DOTATATE is an established treatment option for neuroendocrine tumors (NETs) and has been extended to other somatostatin receptor (SSTR)-expressing tumors. We aimed to determine its efficacy and safety profile in patients with advanced radioiodine-refractory differentiated thyroid carcinoma (DTC).

**Methods:**

Seven radioiodine-refractory DTC patients undergoing at least two cycles of PRRT were included. Patients were subdivided into continuous treatment (defined as sequential application of PRRT; 5/7 (71.4%)) vs. discontinuous treatment (with at least one-year PRRT-free interval; 2/7 (28.6%)). Baseline SSTR PET was analyzed to determine patients’ eligibility for PRRT. Response was assessed by tumor control as defined by stable (± 30.0%) or decreasing (≥ 30.0%) total tumor volume (PET-derived TTV), thyroglobulin (Tg) and RECIST 1.1 criteria.

**Results:**

SSTR PET showed discernible high uptake (maximum standardized uptake values, 10.4 ± 8.6) in metastases, in particular in the skeleton. Continuous PRRT showed variable tumor control (stable disease / response; TTV: 3/5 (60.0%); Tg: 2/5 (40.0%); RECIST 1.1: 3/5 (60.0%)). All patients undergoing discontinuous PRRT exhibited concordant stable disease upon first follow-up and renewed tumor control upon reinitiating PRRT (RECIST 1.1; decreasing TTV and Tg levels). No Common Terminology Criteria for Adverse Events (CTCAE) Grade 3–5 events occured in both groups.

**Conclusion:**

In advanced radioiodine-refractory DTC, PRRT may be beneficial even after treatment interruptions, without major side effects. Given the small cohort and retrospective design, further prospective studies are needed to optimize PRRT strategies in DTC, in particular in a rechallenge scenario.

## Background

Differentiated thyroid carcinoma (DTC) is the most prevalent endocrine malignancy. The most prevalent forms of DTC are papillary (PTC), follicular (FTC) and oncocytic thyroid carcinoma. Its clinical course is typically favorable following surgical intervention and radioiodine therapy. [1–4]. However, a subset of patients develops radioiodine-refractory disease, which significantly limits treatment options and is associated with increased morbidity and mortality ^1^. Even though this is a rare condition with 4–5 new cases per year per million, there is an urgent need for the development of alternative therapeutic strategies to improve disease control and survival [5, 6].

Peptide receptor radionuclide therapy (PRRT) has emerged as a promising therapeutic approach for tumors expressing somatostatin receptors (SSTR) on their cell surface, in particular in neuroendocrine tumors (NETs). The underlying principle of PRRT is the systemic administration of radiolabeled somatostatin analogs, such as Lu-177-DOTA-D-Phe-Tyr3-octreotate (Lu-177-DOTATATE) or Y-90-DOTA-3-Tyr-octreotide (Y-90-DOTATOC), to deliver targeted radiation to tumor cells while sparing normal tissues. The efficacy of PRRT in NETs has been well established, leading to its approval for gastroenteropancreatic NETs [7, 8].

Given the shared expression of SSTRs in a subset of thyroid cancers (TC), particularly poorly differentiated and medullary subtypes, PRRT has been proposed as a viable treatment approach for radioiodine-refractory TC [9–12]. Oncocytic thyroid neoplasms have shown an increased SSTR expression in non-iodine avid lesions [13, 14]. Consequently, SSTR imaging can be used to assess patients’ eligibility for SSTR-directed PRRT [15].

Several retrospective studies and case series have reported promising results regarding the use of PRRT in advanced differentiated TC. Despite the fact that PRRT has been employed since 1999 for this tumor entity, the evidence for its use remains limited [16, 17]. Some studies reported partial remission or disease stabilization in patients with progressive radioiodine-refractory differentiated TC [18]. Versari et al*.* treated 11 patients with differentiated TC with Y-90-DOTATOC. In 71% of patients, disease control was observed (lesion-based analysis), CT morphologic stable disease or partial response was achieved in 60% and 63%, respectively [12]. In patients with medullary TC, different studies showed a decreased lesion size as well as a decreased lesional FDG uptake [15, 19]. Salavati et al*.* reported about stable disease or partial response in 22/28 (78.6%) patients with medullary TC. The incidence of toxicity was low [20]. Lapa et al*.* demonstrated tumor heterogeneity on SSTR PET as a good predictor of response to PRRT in patients with advanced differentiated and medullary TC[21].

However, the number of patients included in these studies, in particular in patients with differentiated TC, remains limited, especially when investigating specific histologic subtypes such as FTC, and further data are needed to define optimal patient selection criteria, treatment protocols, and long-term outcomes. This applies in particular to a discontinuous therapeutic scenario, i.e. to initiate a rechallenge after a minimum of one-year PRRT-free interval.

The primary objective of this study is to evaluate the utility of SSTR PET imaging for 1) PRRT eligibility and 2) therapy monitoring in a case series of PRRT-treated (Lu-177-DOTATATE) DTC patients. Furthermore, the investigation of treatment response based on both, imaging findings and biochemical markers, is intended to facilitate a more profound understanding of the dynamics of PRRT efficacy in this patient population, including in rechallenge setting. By providing additional clinical evidence, we aim to contribute to the growing body of literature on PRRT in thyroid malignancies and explore its potential role as a therapeutic alternative for radioiodine-refractory DTC.

## Material and Methods

### Study Design and Patients

As part of a retrospective observational study at a tertiary cancer center, we included seven patients with histologically proven FTC or oncocytic thyroid carcinoma, who were eligible for PRRT and treated from 2011 to 2024 following the German Medicinal Products Act §13(2b). Written consent was obtained from all patients prior to undergoing PRRT. The analysis was performed in compliance with the principles of the Declaration of Helsinki and was approved by the institutional ethics committee of the Ludwig-Maximilians-University of Munich (IRB #21–0102 and #24–0982). The general patient characteristics included clinical and tumor specific characteristics. Response to treatment was analyzed using PET- and CT- derived results as well as changes in thyroglobulin (Tg) levels. Tg levels were consistently obtained at the time of each staging PET/CT scan—both at baseline and during follow-up assessments.

### Radiopharmaceutical

[^68^ Ga]Gallium was extracted from a Ge-68/Ga-68 generator GalliaPharm® (Eckert&Ziegler AG, Berlin, Germany). [^68^ Ga]Gallium-DOTA-TATE was synthesized using a cassette-based GRP® synthesis module (Scintomics, Fürstenfeldbruck, Germany) and [^68^ Ga]Gallium-DOTA-TOC was synthesized as a kit-formulation (SomaKit®, Novartis Radiopharmaceuticals GmbH, Nuremberg, Germany). Both were injected intravenously at a mean activity of 211.2 ± 48.9 MBq (5.7 ± 1.3 mCi) [22, 23]. All radiolabeling was performed at the department of nuclear medicine according to protocols as describes previously [24].

The Sifalin-TATE (acetate salt) precursor was obtained from ABX Advanced Biochemical Compounds (Radeberg, Germany). Radiosynthesis was performed on a synthesis module GRP® (Scintomics, Fürstenfeldbruck, Germany) or NEPTIS® DB platform (ORA/NEPTIS, Philippeville, Belgium) with high yields and within 30–40 min. All quality controls were performed in accordance with the EuropeanPharmacopoeia. The tracers met the required criteria without exception[25–27]. [^18^F]F-SiTATE was injected intravenously at a mean activity of 207.2 ± 40.9 MBq (5.6 ± 1.1 mCi).

No-carrier added [^177^Lu]Lutetium was obtained from Isotope Technologies Munich S.E. (Garching, Germany) and DOTA-3-iodo-Tyr3-octreotate from att/Scintomics (Fürstenfeldbruck, Germany). Radiolabeling was performed as described elsewhere[28]. All radiopharmaceuticals were administered as infusions of 10 to 50 mL.

### Imaging Protocol

All PET/CT scans were acquired at the Department of Nuclear Medicine at LMU Munich using a Siemens Biograph mCT flow (Siemens Healthineers, Erlangen, Germany). Image acquisition started at a mean of 90.4 ± 21.6 min (F-18) or 68.6 ± 33.8 min (Ga-68) after tracer injection and recorded for 20 min [25]. In the absence of any medical contraindication, patients received furosemide as a premedication for radiation protection and 1.5 mL of iopromide (Ultravist-300, Bayer Healthcare, Leverkusen, Germany) per kg body weight to obtain contrast-enhanced, diagnostic CT scans in the portal-venous phase [29]. Image reconstruction was performed iteratively using TrueX (3 iterations and 21 subsets, 3D Gauss post-filter of 4-mm full width at half maximum). The slice thickness in CT was measured at 0.3 cm.

#### PRRT

Eligibility for PRRT was determined by an interdisciplinary tumor board based on the presence of radioiodine-refractory disease, adequate SSTR expression on PET/CT imaging, and the absence of viable alternative treatment options—such as contraindications to standard systemic therapies or a lack of targetable molecular alterations.

Patients underwent a minimum of two cycles of Lu-177-DOTATATE treatment. Treatment regimens were differentiated between continuous (baseline PET approximately 6 weeks prior to PRRT, two cycles of PRRT at two-month intervals, follow up PET with a mean time interval of 2.2 months; in case of tumor control, another two cycles were added as well as another follow up PET) and discontinuous treatment. The discontinuous regimen consisted of a baseline PET, two cycles of PRRT, and follow up PET. The PRRT was paused until further progression was observed (at least one year; “rechallenge scenario”) [30, 31].

For nephroprotection, co-infusion of amino acids (2.5% Lysin and 2.5% Arginine) was initiated 30 min prior to the administration of Lu-177-DOTATATE. Lu-177-DOTATATE was injected intravenously at a mean activity of 7,482 ± 295 MBq (202 ± 8.0 mCi) in accordance with previous published injection recommendations [32, 33]. In accordance with the German radiation protection regulations, patients were required to remain in hospital for a minimum of 48 h following PRRT. Common Terminology Criteria for Adverse Events (CTCAE) v5.0 were also assessed as described previously [34].

### Patient Characteristics

Seven patients with DTC were included (mean age 70.0 ± 6.9 years; 3 female/4 male). Five of the seven patients were diagnosed with FTC and two with oncocytic thyroid carcinoma (Table 1). Pretreatments are displayed in Table 1. All patients underwent a thyroidectomy and at least one radioiodine therapy, yet no radioiodine uptake was detected at initial diagnosis or tumor lesions lost their ability to trap radioiodine in the course of the disease, despite the presence of metastases as indicated by CT imaging [35]. 1/7 patients underwent cyberknife treatment of cerebral metastases, 4/7 patients underwent radiation therapy (#2: mediastinal metastases; #4: cervical metastases; #5: osseous metastases; #6: thyroid bed and pulmonary metastases). 3/7 patients underwent re-differentiation treatment with either trametinib or rosiglitazone. One patient initially received isotretinoin, followed by rosiglitazone. However, re-differentiation treatment did not result in re-induction of radioiodine avidity in any of the 3 patients. 3/7 patients underwent systemic treatment (#2: lenvatinib; #4: lenvatinib, cabozantinib, pazopanib; #5: sorafenib). Two patients experienced adverse effects, leading to the discontinuation of systemic treatment. One patient received treatment with denosumab.

**Table 1:** General patient characteristics

Pat. ID	Sex	Age	Initial Pathology	Tumor type	Mutations	Pretreatment*	Interval from initial diagnosis/iodine-refractory disease to PRRT [years]		Status of disease prior to PRRT	Regimen	
1	F	74	pT2 pN0 (0/10) M0 R0	follicular	HRAS, BCOR	cyberknife	8/n.a	stable			continuous
2	M	74	n.a	oncocytic	n.a	RT; rediff trametinib; lenvatinib	2/1	stable			continuous
3	F	69	pT3 pN0 (0/9) M0	follicular	n.a		12/9	progressive			continuous
4	M	81	pT3 pN0 (0/8) M0 R1	oncocytic	n.a	RT; lenvatinib; cabozantinib; pazopanib	9/7	progressive			continuous
5	M	64	pT3 pNX pM0	follicular	n.a	RT; rediff rosiglitazone; sorafenib; denosumab	11/3	progressive			continuous
6	M	61	pT1 pNX M1 (PUL)	follicular	n.a	RT; rediff isotretinoin; rediff rosiglitazone	22/4	progressive			discontinuous
7	F	66	pT2 N0 M1 (PUL)	follicular	n.a		4/4	progressive			discontinuous
Mean		70.0									
StD		6.9									

*F* female; *M* male; *n.a.* not available; *rediff* redifferentiation; *RT* radiation therapy; *StD* standard deviation; * all patients underwent thyroidectomy and radioiodine therapy

Following a median period of nine years (range: 2–22 years) since the initial diagnosis, PRRT was initiated.

**Fig. 1:**
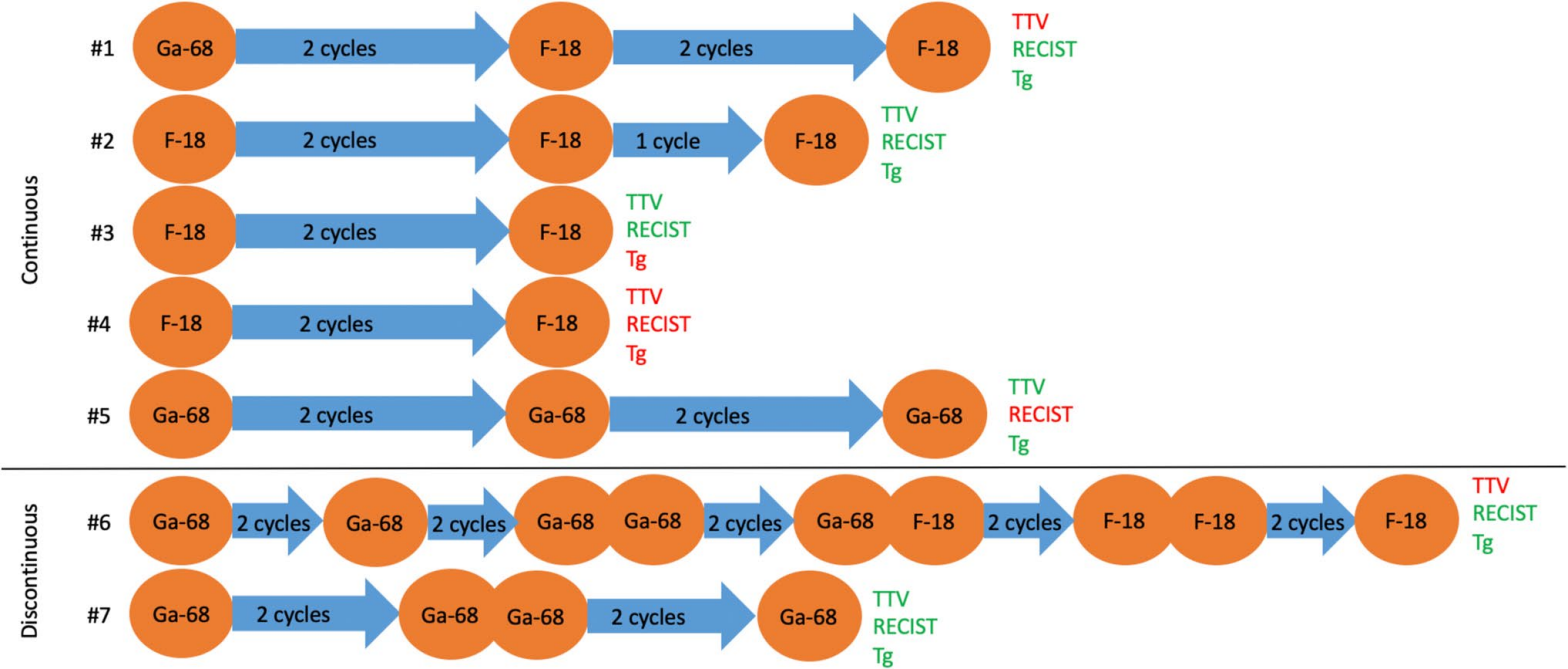
Overview of PET/CT imaging and therapy regimens: Patients #1-#5 received continuous PRRT. 2/5 underwent baseline Ga-68 SSTR PET and two cycles of PRRT, with one patient undergoing Ga-68 (and two further cycles of PRRT) and one patient undergoing F-18 follow up PETs (and two further cycles of PRRT). The other 3/5 patients underwent F-18 PETs only**.** After completion of PRRT 3/5 patients showed tumor control with decreasing or stable TTV, 2/5 showed decreasing or stable thyroglobulin levels and 3/5 stable disease according to RECIST 1.1 criteria. Patients #6-#7 underwent discontinuous PRRT. #6 underwent 10 cycles of discontinuous PRRT, he underwent initially Ga-68 imaging and later F-18 imaging. After four cycles PRRT was paused for eight years, after cycle 6 and 8 for two years, respectively. He presented with tumor control after the 9th and 10th cycle. #7 underwent discontinuous PRRT and Ga-68 imaging only. After two cycles, PRRT was paused for one year. She showed tumor control in all three parameters. *Tg* thyroglobulin; *TTV* total tumor volume; green letters: tumor control; red letters: progression.

5/7 (71.4%) underwent continuous PRRT. Prior to PRRT 2/5 (40.0%) patients presented with stable disease and 3/5 (60.0%) with mild tumor progression. 2/5 (40.0%) patients underwent two cycles, 1/5 (20.0%) patients three cycles and 2/5 (40.0%) patients four cycles. 2/5 (40.0%) patients had a Ga-68 baseline scan, with one patient undergoing Ga-68 and one patient undergoing F-18 follow up PETs. The other 3/5 (60.0%) patients underwent F-18 PETs only (Fig. 1).

Discontinuous PRRT was performed on 2/7 (28.6%) of cases. Both patients presented with slowly progressive disease prior to PRRT initiation. One patient underwent ten cycles (four cycles; eight-year interval receiving sorafenib and radiation therapy of pulmonary metastases; two cycles; two-year interval receiving lenvatinib; two cycles; two-year interval; two cycles). All tyrosine-kinase-inhibitor therapies were ceased due to severe side effects.

As the other patient exhibited a low tumor volume and gradual progression over a period of three years, there was no indication for tyrosine-kinase-inhibitor therapy. She received four cycles (two cycles and after one year another two cycles). One patient underwent Ga-68 scans only, the other one underwent five Ga-68 scans followed by four F-18 scans (Fig. 1).

### Image Analysis

A dedicated software package was used for PET analysis (Hermes Hybrid Viewer, Affinity 1.1.4; Hermes Medical Solutions, Stockholm, Sweden). A single lesion analysis (maximum standardized uptake values (SUV_max_)) was conducted to assess PRRT eligibility. Localizations were categorized into: metastasis (nodal, osseous, visceral, other) and local recurrence. The tumor-to-background ratio was determined visually by applying the Krenning score on the lesion with the highest and lowest SSTR expression. No uptake was defined as 0, very low uptake as 1, uptake less than or equal to the liver as 2, uptake greater than the liver as 3 and uptake greater than the spleen as 4 [17]. The PET-based treatment response was assessed using total tumor volume (TTV; stable ± 30.0%; decrease −30.0%; increase + 30.0%). TTV was determined in a semiautomated manner, applying a fixed SUV threshold of 4.0 (F-18) and an individualized threshold (Ga-68), as described previously [11, 36].

The evaluation of CT datasets was performed according to RECIST 1.1 on a dedicated software (mint lesion™, version 3.8.6, Mint Medical GmbH, Dossenheim, Germany) [37, 38].

Parameters derived from the imaging were compared with Tg values at the same time point. Tumor control was defined as stable (± 30.0%) or decreasing (−30.0%) TTV or Tg or stable disease (SD) according to RECIST 1.1.

Patients underwent at least a baseline and one follow up PET/CT imaging after two cycles of PRRT.

### Statistical Analysis

Data analysis was performed in a descriptive manner using Microsoft Excel (Excel 2019, Microsoft, Redmond, WA, USA). Statistics are displayed as mean ± standard deviation (StD).

## Results

### Single Lesion Analysis on Baseline PET

#### Ga-68-Based Tracers

A total of 177 single lesions in four patients on Ga-68 baseline SSTR PET were analyzed (Table 2). The highest uptake was detected in osseous metastases with a mean SUV_max_ of 9.2 ± 3.4 as well as in one soft tissue metastasis of the thorax with a SUV_max_ of 9.9. On an individual lesion level, the highest Krenning score in all patients was 3 (4/4 100%).

**Table 2:** Ga-68 SSTR PET: Single lesion analysis (SUV_max_)

No. of patients			Nodal (n = 7)	Osseous (n = 12)	Visceral (n = 27 lung)	Other (n = 1)	Recurrence
4		Mean	6.9	9.2	6.5	9.9	n.a
		StD	1.7	3.4	3.3	n.a	n.a

*n.a.* not applicable; *StD* standard deviation

### F-18-SiTATE

A total of n = 61 single lesions in three patients on F-18-SiTATE baseline PET were analyzed (Table 3). The highest mean SUV_max_ was seen in one osseous metastasis (19.7) and other (muscular) metastases (33.9 ± 21.7). Furthermore, one local recurrence with a SUV_max_ of 27.1 was detected. On an individual lesion level, the highest Krenning score was 4 in one patient, 3 in one patient and 2 in one patient. SSTR expression was considered sufficient for PRRT.

**Table 3:** F-18-SiTATE PET: Single lesion analysis (SUV_max_)

No. of patients			Nodal (n = 7)	Osseous (n = 1)	Visceral (n = 3 lung, n = 1 liver, n = 1 kidney)	Other (n = 2)	Recurrence (n = 1)
3		Mean	16.5	19.7	16.6	33.9	27.1
		StD	9.8	n.a	12.7	21.7	n.a

*n.a.* not applicable; *StD* standard deviation

### Response to PRRT

#### Continuous Treatment

**Fig. 2:**
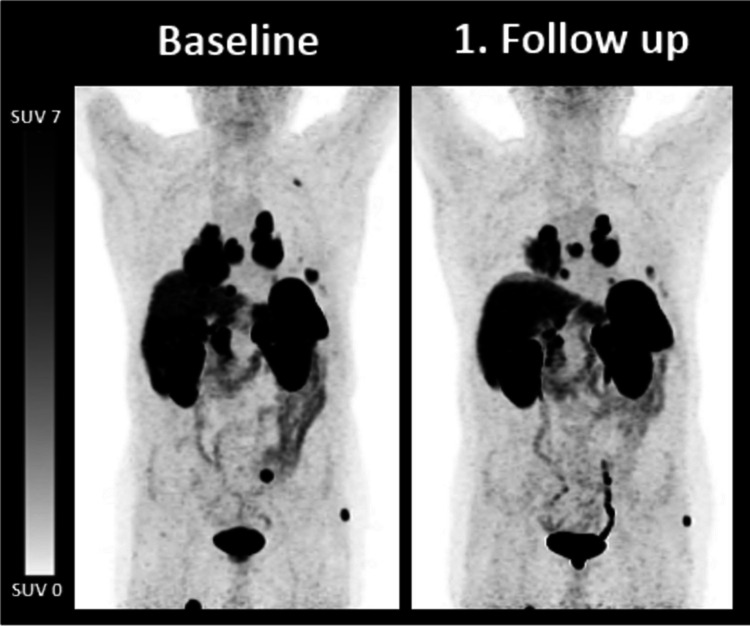
MIP of patient #4: The 30-year-old male patient was diagnosed with oncocytic thyroid carcinoma and underwent continuous PRRT. The baseline F-18-SITATE PET showed a TTV of 191 mL. After two cycles of PRRT, the follow up F-18 PET showed an increased TTV 400 mL. The thyroglobulin level increased from 19135 ng/mL to 25085 ng/mL

2/5 (40.0%) patients presented with stable disease and 3/5 (60.0%) with mild tumor progression prior to PRRT. Patient #1 and #4 presented with an increase of TTV. According to RECIST 1.1, patient #1 was defined with SD and #4 with progressive disease (PD) due to new lesions. Tg levels were stable in #1 and increasing in #4 (Table 4; Fig. 2).

**Table 4:** Biochemical and imaging parameters in patients with continuous regimen

	BL			1. FU				2. FU			
Pat. ID	Tracer	TTV (mL)	Tg (ng/mL)	Tracer	TTV (mL)	RECIST 1.1	Tg (ng/mL)	Tracer	TTV (mL)	RECIST 1.1	Tg (ng/mL)
1	Ga-68	3.9	0.2	F-18	41.0	SD	0.6	F-18	50.3	SD	0.2
2	F-18	385.0	844	F-18	586.0	SD	586	F-18	233.0	SD	449
3	F-18	10.1	778	F-18	10.6	SD	1058				
4	F-18	191.0	19,135	F-18	400.0	PD	25,085				
5	Ga-68	91.4	25,000	Ga-68	90.9	SD	37,509	Ga-68	117.0	PD	46,844

*BL* baseline; *FU* follow up; *PD* progressive disease; *SD* stable disease; *Tg* thyroglobulin; *TTV* total tumor volume

Patient #2 presented with an increase of the TTV between baseline and follow up 1 (385 mL vs. 586 mL) but decrease at follow up 2 (233 mL). RECIST 1.1 criteria defined the patient with SD, Tg levels were decreasing over the course (Table 4).

Patient #3 presented with a stable TTV and SD despite increasing Tg levels (Table 4).

Patient #5 presented with stable TTVs from baseline to follow up 2. According to RECIST 1.1 this patient was defined as SD at follow up 1 and PD at follow up 2 and Tg levels increased accordingly (25,000 ng/mL vs. 37,509 ng/mL vs. 46,844 ng/mL) (Table 4).

**Fig. 3:**
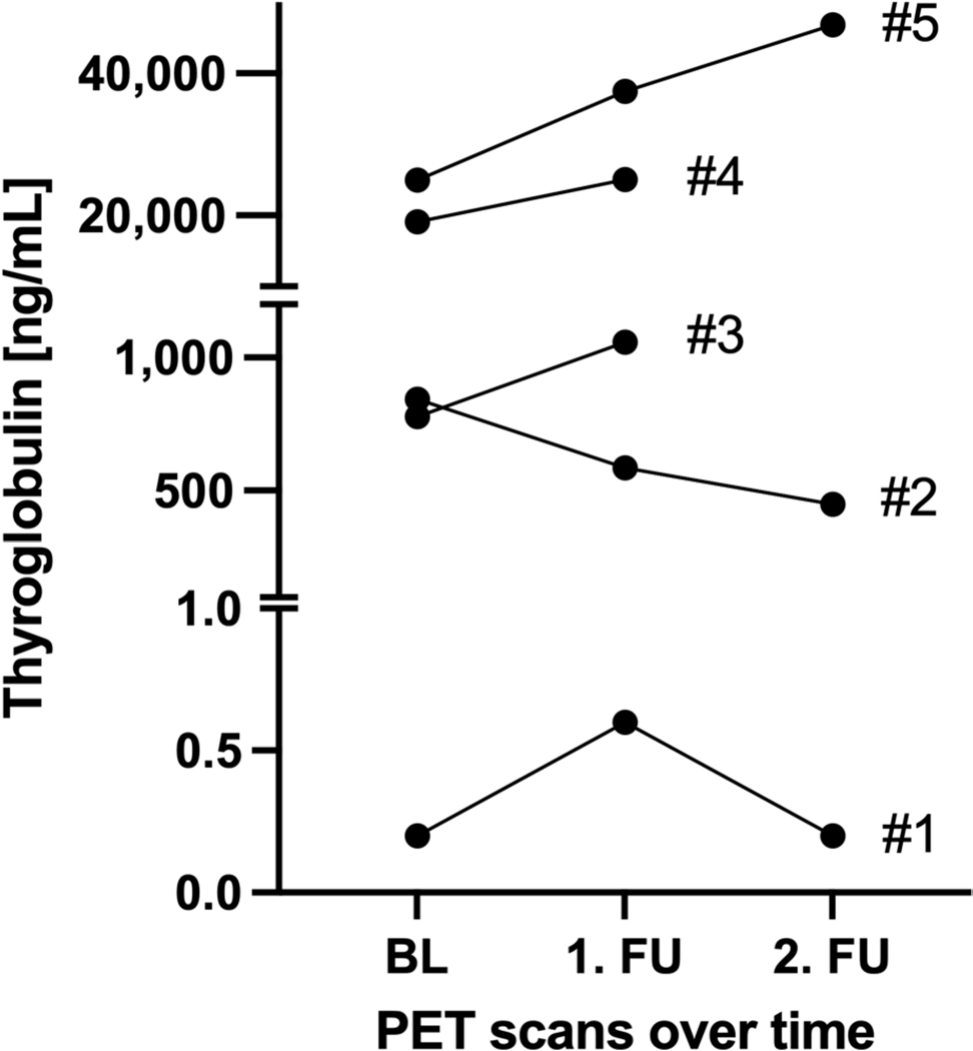
Thyroglobulin levels in patients undergoing continuous PRRT: Thyroglobulin levels for patients #1-#5 are displayed for baseline, follow up 1 and follow up 2 SSTR PET

After completion of PRRT, 3/5 (60.0%) patients showed tumor control in TTV, 2/5 based on Tg levels (40.0%; Figs. 1 and 3) and 3/5 (60.0%) based on RECIST 1.1 criteria. Except patient #2, imaging and biochemical parameters showed heterogenous responses to PRRT (Table 4; Fig. 1).

### Discontinuous Treatment

**Fig. 4:**
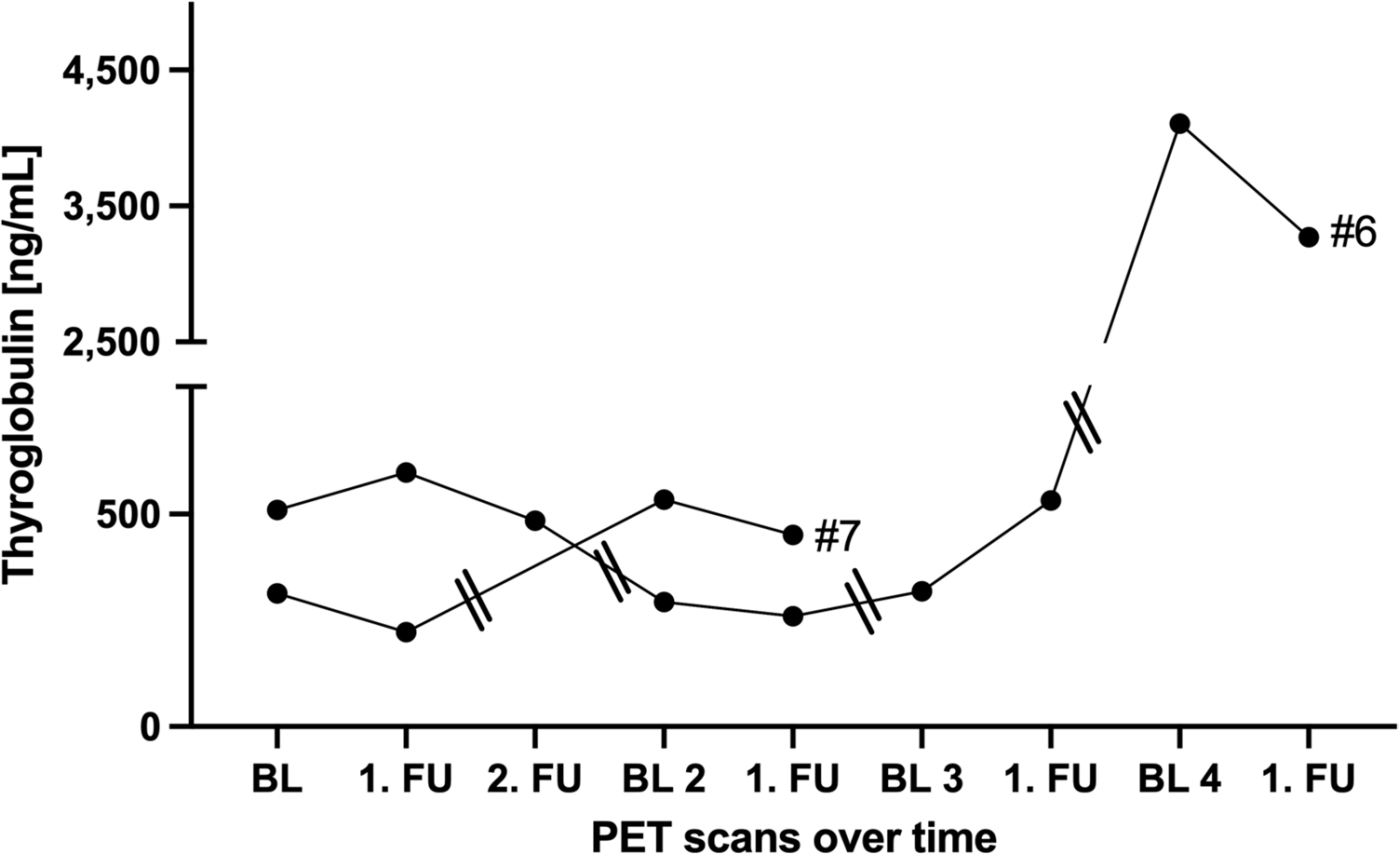
Thyroglobulin levels in patients undergoing discontinuous PRRT: Longitudinal course of thyroglobulin levels over time for patients #6-#7 aligned with PET imaging time points (baseline (BL), follow up (FU)). A break of at least one year is indicated by a double vertical line

Both patients, who underwent discontinuous treatment, presented with slowly progressive disease prior to PRRT initiation. Patient #6 underwent ten cycles of PRRT. This individual showed mainly consistent imaging and biochemical tumor control. During PRRT-free intervals, Tg levels increased. After completion of the final cycle 10, the subject presented with a heterogenous response with increasing TTV, but decreasing Tg (Table 5; Figs. 1 and 4).

**Table 5:** Biochemical and imaging parameters in patients with discontinuous regimen

Pat. ID	BL 1 prior to cycle 1—4			1. FU			
	Tracer	TTV (mL)	Tg (ng/mL)	Tracer	TTV (mL)	RECIST 1.1	Tg (ng/mL)
6	Ga-68	4.6	510	Ga-68	3.5	SD	598
	BL 2 prior to cycle 5—6			1. FU			
	Tracer	TTV (mL)	Tg (ng/mL)	Tracer	TTV (mL)	RECIST 1.1	Tg (ng/mL)
	Ga-68	1.5	293	Ga-68	0.8	SD	260
	BL 3 prior to cycle 7—8			1. FU			
	Tracer	TTV (mL)	Tg (ng/mL)	Tracer	TTV (mL)	RECIST 1.1	Tg (ng/mL)
	F-18	3.3	318	F-18	5.5	SD	532
	BL 4 prior to cycle 9—10			1. FU			
	Tracer	TTV (mL)	Tg (ng/mL)	Tracer	TTV (mL)	RECIST 1.1	Tg (ng/mL)
	F-18	27.9	4,105	F-18	73.6	SD	3272
Pat. ID	BL 1 prior to cycle 1—2			1. FU			
	Tracer	TTV (mL)	Tg (ng/mL)	Tracer	TTV (mL)	RECIST 1.1	Tg (ng/mL)
7	Ga-68	10.9	313	Ga-68	9.5	SD	222
	BL 2 prior to cycle 3—4			1. FU			
	Tracer	TTV (mL)	Tg (ng/mL)	Tracer	TTV (mL)	RECIST 1.1	Tg (ng/mL)
	Ga-68	34.0	534	Ga-68	3.8	SD	451
Pat. ID	2. FU						
	Tracer		TTV (mL)		RECIST 1.1	Tg (ng/mL)	
6	Ga-68		3.7		SD	485	

*BL* baseline; *FU* follow up; *SD* stable disease; *Tg* thyroglobulin; *TTV* total tumor volume

**Fig. 5:**
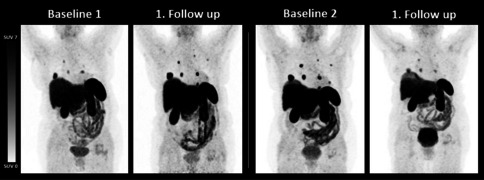
MIP of patient #7: The 66-year-old female patient was diagnosed with FTC and underwent discontinuous PRRT. The initial baseline Ga-68 SSTR PET showed a TTV of 10.9 mL. After two cycles of PRRT, TTV slightly decreased to 9.5 mL. The thyroglobulin level decreased from 313 ng/mL to 222 ng/mL. RECIST 1.1 analysis revealed stable disease (SD). After an interval of one year, the patient underwent a baseline Ga-68 SSTR PET prior to another two cycles of PRRT. After these two cycles, TTV decreased from 34 mL to 3.8 mL (RECIST 1.1: SD). The thyroglobulin level decreased from 534 ng/mL to 451 ng/mL

Patient #7 underwent four cycles of PRRT and responded to PRRT with a decrease of TTV and Tg (Table 5; Figs. 1, 4 and 5).

As such, to sum up, both subjects always exhibited SD according to RECIST 1.1 upon the first treatment series of treatment cycles and upon rechallenge, which was further accompanied by declining Tg levels.

### Safety Profile

There were no CTCAE Grade 3–5 adverse events reported in patients of both regimens. For continuous treatments, we observed CTCAE Grade 1–2 events in all five patients (1: 3/5 (60.0.%); 2: 2/5 (40.0%)), while only in one of two patients with discontinuous treatment CTCAE Grade 2 was reported.

## Discussion

The findings of this case series indicate that SSTR PET imaging is a valuable tool for 1) assessing patients´ eligibility for PRRT and 2) monitoring the response to PRRT in patients with DTC. Notwithstanding the heterogeneous nature of the patient cohort, the results indicate that serial SSTR PET scans provide meaningful insights into disease progression and treatment response. A response to PRRT was seen in both sub cohorts, patients with continuous and discontinuous regimen. Of note, the latter group always exhibited SD following RECIST 1.1, while Tg levels decreased even after a one-year PRRT-free interval, thereby indicating that rechallenge may be a useful therapeutic approach even in radioiodine-refractory DTC.

In relation to the response to treatment, highly variable courses were observed among patients undergoing continuous PRRT regimens. While some patients demonstrated SD or PR, others exhibited mixed responses with discordant findings across imaging modalities and biochemical markers. This discrepancy indicates that neither imaging-based nor laboratory-based parameters alone are sufficient to comprehensively evaluate treatment efficacy. The absence of a discernible pattern among the assessed parameters underscores the intricacy of disease monitoring in this context and suggests the necessity of a multimodal assessment approach.

It is noteworthy that, in the subgroup of patients following a discontinuous PRRT regimen, disease remained stable over the course of several years. Upon subsequent progression, the re-initiation of PRRT frequently resulted in a renewed treatment response, indicating that PRRT maintains efficacy even following treatment interruptions of more than one year. This finding is consistent with the results of previous studies in neuroendocrine tumors, where PRRT retreatment has also demonstrated clinical benefits [31, 39]. In this regard, discontinuous PRRT may contribute to “disease chronification” in radioiodine refractory DTC until the next escalation in therapeutic management is required. Notably, patients receiving discontinuous treatment generally exhibited smaller tumor volumes, slower disease progression, and had not yet undergone systemic therapy, positioning them earlier in the treatment sequence. This allowed for a more flexible treatment regimen. This approach was consciously chosen to balance therapeutic efficacy with potential toxicity. However, in cases of rapid progression and extensive tumor burden, a discontinuous regimen may not be suitable, as delays in treatment could compromise disease control. These findings emphasize the need for individualized therapy decisions, considering both tumor kinetics and patient-specific factors to optimize treatment outcomes.

The heterogeneity of the patient cohort is a significant limitation, as the diversity in metastatic patterns (visceral, pulmonary, osseous and other metastases) may influence treatment response. For instance, patients who received a continuous PRRT regimen predominantly exhibited mixed visceral metastases, while those who received discontinuous treatment exhibited primarily pulmonary metastases. A different burden of osseous metastases was also detected between the subgroups. Only one patient received denusomab and radiation therapy of the bone. These observations suggest that metastatic distribution and the presence of locoregional recurrence may play a role in PRRT response, warranting further investigation in larger, more homogenous cohorts.

Since the Krenning score was originally developed for scintigraphy using In-111-pentetreotide SPECT in NET patients, an additional single lesion analysis was conducted for baseline PET/CTs to validate the visual assessment [17]. It should be noted, however, that the Krenning score has not yet been validated for DTC.

The methodology of Ga-68 SSTR PET imaging analysis was based on the delineation approach described by Seifert et al*.*, which was originally developed for prostate cancer and not explicitly validated for thyroid cancer ^36^. Notwithstanding this limitation, our visual assessments demonstrated reasonable lesion segmentation, with high uptake values in single-lesion analysis allowing for clear delineation from background activity [17]. The ability of PET imaging to detect new lesions supports its utility in treatment monitoring, even in the absence of a validated thyroid cancer-specific PET scoring system. Furthermore, discrepancies between PET and CT findings were noted in several patients, raising the question of whether these differences impact long-term prognosis – a topic requiring further exploration.

The present findings are consistent with those of earlier studies reporting variable responses to PRRT in thyroid malignancies [15, 18, 40]. While some retrospective analyses have demonstrated favorable response rates, others have emphasized the challenge of predicting PRRT efficacy based on standard imaging or laboratory markers [12, 41]. Additionally, the role of whole body SUV_max_ as predictive factor for PRRT response has not been conclusively clarified for NETs and therefore even less for DTC [42]. Thus, this was explicitly not reported in this study. The complexity of disease progression in radioiodine-refractory differentiated thyroid carcinoma necessitates the refinement of response assessment criteria and the identification of novel biomarkers that may more accurately predict treatment outcomes. Potential biomarkers include quantitative measures of somatostatin receptor expression derived from PET imaging, circulating tumor DNA and molecular mutational profiles, as well as immunohistochemical markers reflecting tumor microenvironment and immune status. Advanced imaging features such as radiomics and texture analysis from PET/CT could further enhance patient stratification by capturing tumor heterogeneity and metabolic activity. Integrating these biomarkers with clinical parameters may improve the selection of patients most likely to benefit from PRRT and guide personalized treatment strategies in future studies [43–45].

In general, PRRT was well tolerated in both cohorts, which is line with previous results [46]. Newer targeted systemic therapies for DTC are available but were not available at the time the study cohort mainly underwent PRRT. Unlike systemic therapies, PRRT is associated with fewer side effects than systemic therapies; however, the lack of large comparative trials between PRRT and systemic therapies in TC patients means that the value of PRRT in DTC needs to be further investigated [47, 48].

A key limitation of our study is the small cohort size, which limits the generalizability of our findings. Due to the small cohort, it was not differentiated between FTC and oncocytic thyroid carcinoma [49]. Both patients with oncocytic thyroid carcinoma underwent re-differentiation therapy as well as radiation therapy of either a mediastinal tumor mass or cervical metastases. One patient showed a stable tumor burden (#2) prior to PRRT, whereas the other patient (#4) showed progressive disease under lenvatinib. Interestingly, one patient #2 responded well to PRRT, whereas the #4 presented with progressive disease. Consequently, the progression of the disease prior to PRRT may also have an impact on the response to PRRT. Additionally, the retrospective study design introduces potential biases, such as patient selection and data availability. A lesion-based analysis was not performed in this study, as the limited number of patients would have resulted in a very small number of metastatic lesions, making a meaningful evaluation difficult. Additionally, the imaging protocols varied, with different tracers being used, which complicates direct comparisons between patients. Furthermore, the number of follow-up PET/CT scans was highly heterogeneous. These factors introduce variability that limits the comparability of individual treatment responses and should be considered when interpreting the results. The patients included in this study exhibited a wide range of clinical statuses at the time of PRRT initiation, with some presenting with stable disease and others with progressive disease. Additionally, most of the patients had long courses of their disease with active surveillance after failure of radioiodine therapy, and in part also sequential multimodal pretreatments, including systemic therapies with multityrosine kinase inhibitors and redifferentiation approaches. This heterogeneity may have influenced treatment response and should be considered with caution when interpreting the findings.

Although based on a small cohort of only seven patients, PRRT appears to be a generally well-tolerated treatment option that may help to slow disease progression in radioiodine-refractory thyroid carcinoma, particularly in patients without targetable mutations. However, further studies with larger populations are necessary to confirm these observations before firm conclusions can be drawn regarding its clinical value compared to other therapies with potentially higher toxicity profiles.

Consequently, these findings should be interpreted with caution. Validation and refinement of therapeutic strategies in this complex patient population are imperative and require the undertaking of prospective studies with larger patient cohorts.

## Conclusion

In summary, the present case series highlights the potential of SSTR PET imaging and Tg levels as a valuable tool for monitoring PRRT response in patients with DTC, despite the variability in PET tracers. While treatment responses were found to be heterogeneous, the study suggests that PRRT retains efficacy even after treatment interruptions, while safety profile was acceptable (no CTCAE Grade ≥ 3). In light of the study's limitations further prospective research with larger patient populations is necessary to refine patient selection criteria and optimize PRRT strategies in DTC, including rechallenge scenarios.

## Data Availability

The datasets used and/or analyzed during the current study are available from the corresponding author on reasonable request.

## Abbreviations

BL: Baseline
CTCAE: Common Terminology Criteria for Adverse Events
DTC: Differentiated thyroid carcinoma
F: Female
FU: Follow up
FTC: Follicular thyroid carcinoma
M: Male
n.a.: Not applicable
NET: Neuroendocrine tumor
PD: Progressive disease
PRRT: Peptide receptor radionuclide therapy
PTC: Papillary thyroid carcinoma
RT: Radiation therapy
StD: Standard deviation
SD: Stable disease
SSTR: Somatostatin receptor
SUV_max_: Maximum standardized uptake value
Tg: Thyroglobulin
TTV: Total tumor volume
